# Teaching Medical Students Optimal Consulting Skills: The Challenge of Generating Better Referring Physicians

**DOI:** 10.7759/cureus.5172

**Published:** 2019-07-18

**Authors:** Andrew W Phillips, Terence Potter, Brittany Z Dashevsky, Nicholas Masse, Brent Greenberg, Christopher M Straus

**Affiliations:** 1 Emergency Medicine, University of North Carolina, Chapel Hill, USA; 2 Emergency Medicine, Creighton University Maricopa Medical Center, Phoenix, USA; 3 Radiology, Redwood Radiology Group / St. Joseph Health, Santa Rosa, USA; 4 Breast Imaging, The University of Chicago Medicine, Chicago, USA; 5 Radiology, The University of Chicago Medicine, Chicago, USA; 6 Nuclear Medicine, Thoracic Imaging, The University of Chicago Medicine, Chicago, USA

**Keywords:** medical student curriculum, medical education, radiology clerkship

## Abstract

Rationale and objectives

We sought to incorporate a new teaching module into the traditional medical student radiology clerkship, to improve the necessary skills for future referring physicians.

Materials and methods

A new required and graded module was introduced in 2014 into the radiology clerkship in year three of medical school: the Mystery Case. Each student was provided a unique and undifferentiated case from a dedicated teaching file containing de-identified images and requisition data. Students were expected to complete three serial tasks over one week: 1) prepare a voice recognition-derived, structured radiological report utilizing appropriate and relevant vocabulary; 2) discuss pertinent additional clinical information; and 3) discuss appropriate follow-up imaging, in addition to information on how to best prepare patients for these potential patient exams (e.g., with or without contrast, bowel preparation, and length of study). Students were provided written examples and dedicated class instruction with interactive discussions covering specific cases and associated related cases through random pairing with radiology resident and attending mentors. At the close of the week, students gave brief oral presentations of their cases and submitted the tasks for a written evaluation. Upon completion of the clerkship, the students completed a Likert-type six-item survey to evaluate the perceived improvement in select skills.

Results

The survey was completed by 82% (54/66) of the enrolled students, with 85% finding the Mystery Case an effective use of time. Medical students perceived an improved awareness of the patient care process (77%), awareness of the medical imaging resources available (89%), ability to understand a radiology report (74%), and ability to advise patients (69%).

Conclusion

Introduction of the Mystery Case as a graded exercise in the medical school radiology clerkship was perceived by students as effective use of time, with an improvement in the skills essential for future referring physicians.

## Introduction

The vast majority of medical students (95%) go on to become referring emergency medicine, primary care, or sub-specialty physicians, rather than radiologists (5%), according to the 2017 Association of American Medical Colleges (AAMC) All Schools Annual Report [1]. Since referring physicians initiate most medical imaging, it is essential during medical school to prepare students for this future role with pertinent training. This includes appropriate exam ordering, consideration of resource allocation, and appropriate screening and preparation of patients for imaging studies. These skills are in addition to the effective incorporation of imaging reports into patient care [2]. Although report interpretation skills are essential, in any given year, it is, at most, reported that less than 25% of US medical schools offer radiology training by radiologists, and these experiences are taken as an elective or incorporated into other required clerkships diminishing the overall number of students even further [3]. Additionally, there is no standard radiology curriculum adhered to nationally in the United States [4-5].

To improve radiology education, the Alliance of Medical Student Educators in Radiology (AMSER) developed a radiology curriculum guide for medical schools [6], as well as a standardized web-based exam [7-8]. The establishment of the American College of Radiology (ACR) appropriateness criteria (AC) has also improved imaging utilization. However, these criteria must be reinforced within the medical school curriculum [9].

The quality of the referring physician’s order, the information relayed to the patient when scheduling an imaging exam, and the interpretation of the radiology report, ultimately impact patient care [10-14]. While these skills are taught during radiology residency, it is the referring physician who more often places the imaging order and relays information to the patient. Minimal research in this educational area has been conducted.

We sought to develop an exercise, the Mystery Case, within the medical school required radiology clerkship at the University of Chicago to augment the practical needed skills fundamental to a medical student's role as a future referring physician.

## Materials and methods

In 2014, a new, required, and graded module was introduced into the radiology clerkship, which is a one-week period during the internal medicine rotation of the medical school third-year curriculum. This multi-day exercise began with each student receiving a unique radiology Mystery Case chosen at random from a predetermined file of studies created by faculty radiologists. The pool of cases was selected by a senior experienced student educator, pulling examples from vetted lists of cases and pathology determined and identified as core knowledge. Additionally, the AMSER “Must See” list of images in their curriculum were all represented in the pool of cases [6]. Randomization was performed by students choosing a case on a folded slip of paper from a jar. These studies represented the pathology an emergency medicine, primary care, or sub-specialty physician would be expected to see during any given day of practice. Each student was provided the anonymous images and correlated clinical history from the original exam order.

Over four days, students were required to complete three serial tasks, as follows: 1) generate a voice recognition-derived radiology report that demonstrates an understanding of structured reporting using relevant vocabulary as it pertains to their assigned random case, 2) identify and relay pertinent additional clinical information, which would have been helpful if provided in the original study request, and 3) discuss what follow-up imaging (if any) should then be considered from the perspective of a referring physician, with basic knowledge of potential, related, specialized instructions needed for the patient to be properly prepared (e.g., intravenous (IV) contrast or bowel preparation).

The randomly assigned cases represented patient scenarios that would routinely require advanced follow-up imaging, and most required subsequent procedures, often routinely performed by radiologists. Examples of these cases included, but were not limited to, a screening mammogram with an obvious breast mass, a chest radiograph with a solitary nodule, an abdominal radiograph with bowel obstruction, and a head CT with hemorrhage.

Students were provided written examples and had dedicated class time with interactive discussions of additional cases. The students were given an intervening day to conduct independent research on their unknown case and to generate a draft radiology report highlighting the clear use of structured reporting and standard American College of Radiology key vocabulary tied to the identified pathology. The students were paired one-on-one with a radiology resident mentor who was in his or her second or third year of training, who would assist in generating the report. This resident would have had hundreds of hours of dictation and experience to share and relate, answering questions and providing insights. The students used a provided template to assist them in creating their structured report and had additional templates available to best match their case type.

On the second or third day, the students met in a small discussion group (five to 10 students) with a senior attending radiologist who would reinforce the thought process and medical reasoning required to generate patient-specific individualized imaging, linking and demonstrating directly the influence of the primary clinical information. Algorithmic, first-order thinking, such as a single best answer from a chief complaint, was discouraged during these sessions. The examples reviewed were identical in layout and complexity to the assigned cases, but the specific details of students’ assigned cases were not discussed, leaving the students to generate independent exam interpretation and subsequent workup.

The fourth day consisted of an optional question and answer session with one of the radiology residents, allowing time to absorb information, draw on prior experiences and material presented during the week, and pose any final questions. The final day of the exercise consisted of individual presentations of the Mystery Case to the entire group, allowing students to share specifics and the medical reasoning needed to complete the case. A radiology attending and resident graded each student.

**Figure 1:**
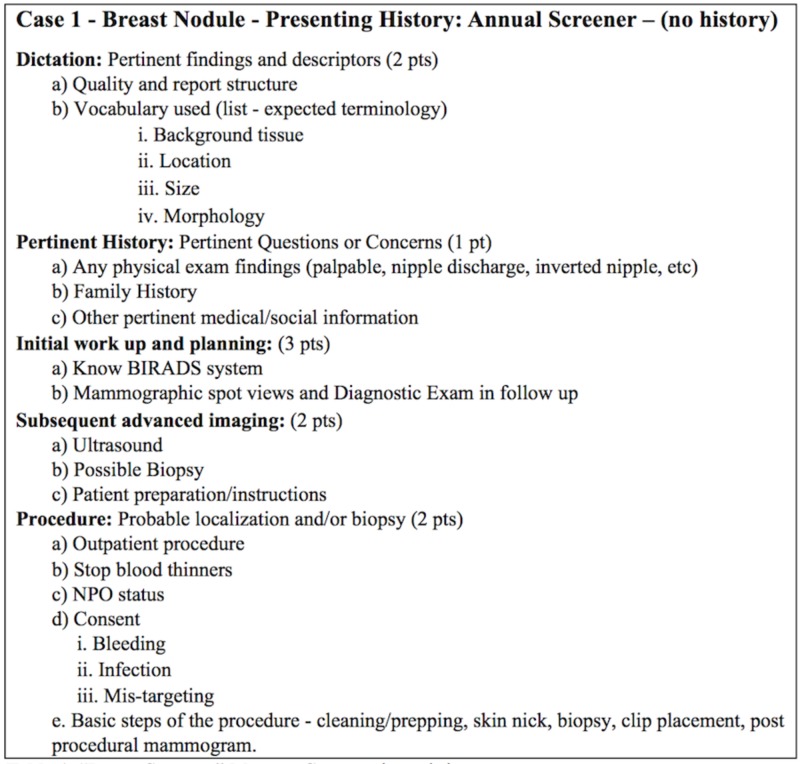
“Breast Screener” Mystery Case scoring rubric

Student scoring followed a standardized rubric, minimally modified to reflect the specifics associated with each case, with one such example in Figure 1 depicting our “Breast Screener” case. As part of the evaluation, students were scored on multiple aspects of their performances, and students could receive a pre-set maximum number of points for each of the designated sections. Graded elements included a structured report demonstrating image interpretation as well as additional comments/understanding of the value that additional pertinent clinical information had on the case. The former highlights the use of key vocabulary and observational skills, and the latter begins to address the often-misunderstood influence that the ordering physician has on image interpretation [10-13].

**Figure 2:**
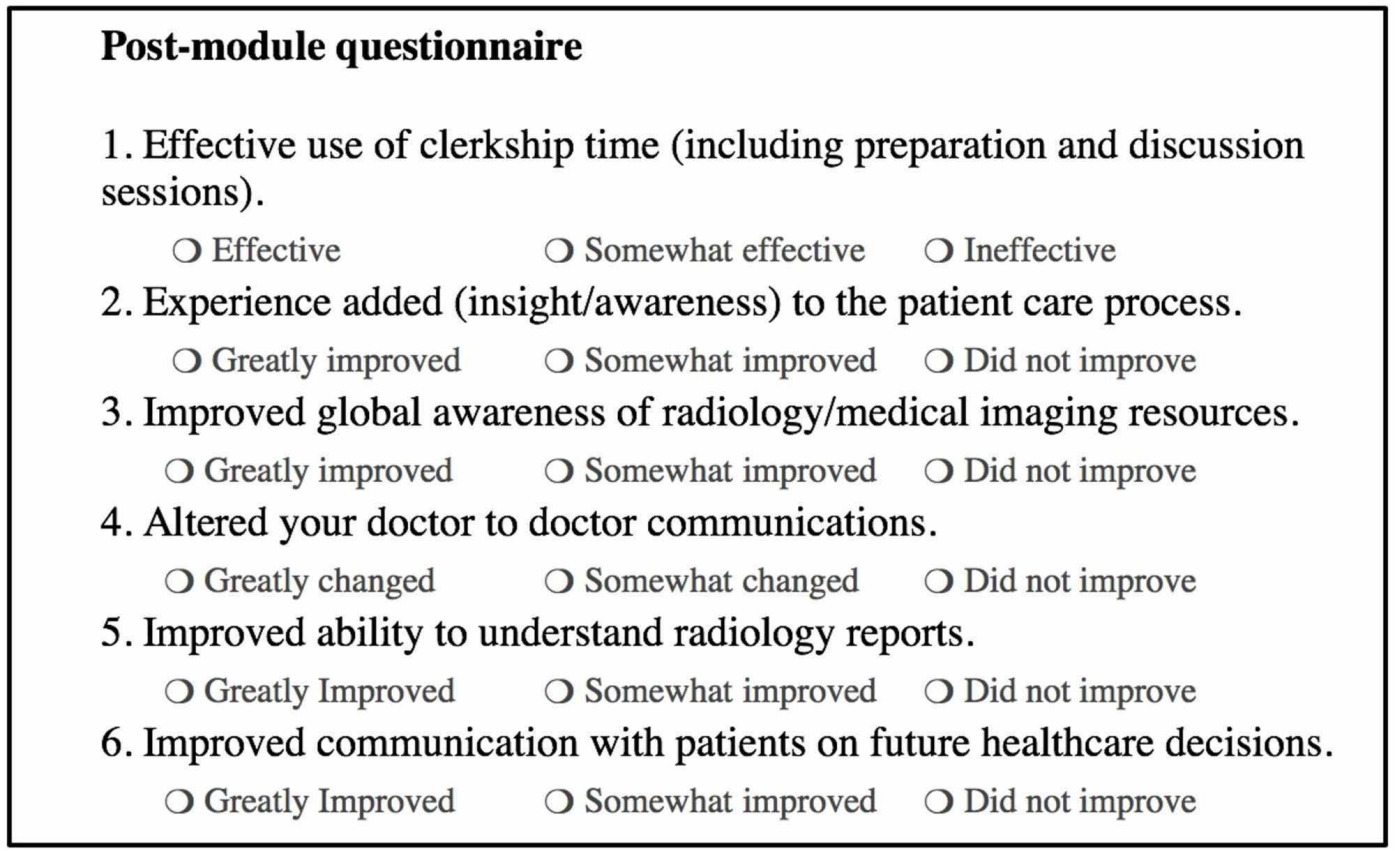
Likert survey administered to medical students following completion of the Mystery Case

A survey consisting of six Likert-type items (Figure 2) was created to gauge how well this module was received by medical students. Experts in radiology and survey design created and evaluated the instrument for construct validity, which was then pilot tested among recent graduate interns. This survey was administered at the conclusion of the teaching module and consisted of six questions asking the students to grade aspects of the experience such as improved ability to understand radiology reports, with a choice of answering very positive, somewhat positive, or no change.

Statistical analysis

Likert data were treated as ordinals for all comparisons, which included chi-square and Spearman’s rho as appropriate. All comparisons assumed α<0.05 for significance and were two-tailed. No Bonferroni adjustment was required since fewer than 10 comparisons were made between variables. All results were collected using Survey Monkey (SurveyMonkey Inc.), entered into Microsoft Excel™ (Microsoft Corporation, Washington, US), and analyzed using SPSS version 21 (IBM Corporation, Armonk, NY, US).

## Results

**Figure 3:**
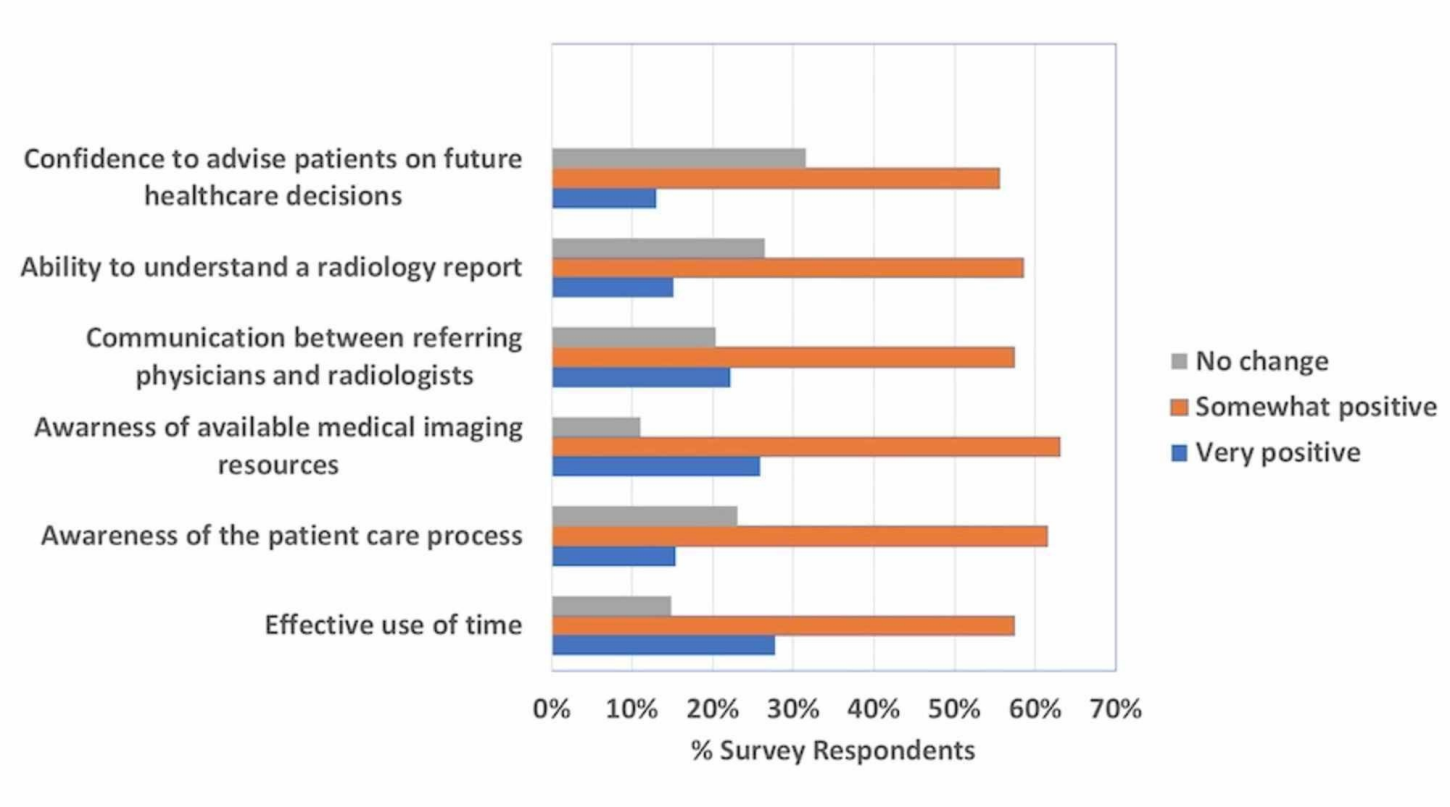
Likert survey results evaluating the Mystery Case module

Of the 66 eligible participants, 54 responded to the survey, providing a response rate of 81.8%, which is the American Association of Public Opinion Research response rate definition number six [15]. Of the 54 respondents, 46.3% were male. Survey results are summarized in Figure 3 and detailed below. Overall, students found the module beneficial.

Eighty-five point two percent (85.2%) found the Mystery Case intervention to be an effective use of their radiology clerkship time. This did not vary significantly between academic quarters $x^{2}(4)=.963, \mathit{p}=.915$, or by sex $x^{2}(2)=1.87, \mathit{p}=.393$, respectively. A full 88.9% reported somewhat or greatly improved awareness of available medical imaging resources to requesting physicians as compared to 76.9% who reported somewhat or greatly improved awareness of the patient care process, which was significantly different, $x^{2}(4)=17.7, \mathit{p}=.001$.

The intervention changed expectations for communications between requesting physicians and radiologists for 79.6% of the participants. Improved communication expectations, however, were not related significantly to students reporting improved ability to understand radiology reports (73.6% reporting some or great improvement), $r_{s}=.187,p=.180$. Finally, 55.6% and 13% reported “somewhat” and “greatly” improved confidence to advise patients on future healthcare decisions, respectively.

Improved awareness of the patient care process had the strongest relationship with our primary question of the effective use of time in the clerkship $r_{s}=.649, p&lt;.001$. An unexpected, but logical, relationship was seen between improved ability to understand radiology reports and improved confidence to advise patients on their future healthcare decisions $r_{s}=.598, p&lt;.001$. In addition to the significant and promising objective data detailed above, anecdotal reports from radiology attending physicians and residents were overall positive.

## Discussion

At the University of Chicago, we implemented the Mystery Case module for medical students during their radiology clerkship in the third year of medical school, designed to reinforce important global knowledge and skills applicable to future referring physicians. Likert survey results demonstrated a perceived improvement in awareness of the patient care process, awareness in available medical resources, understanding of radiology reports, and confidence in advising patients while also changing their expectation of communication between requesting physicians and radiologists.

This educational module utilizes an interactive format to address skills beyond image interpretation while also to set in context to demonstrate potential relevance. To focus a learning experience explicitly on the skills needed by the future referring physician only highlights how radiologist-led exercises and education can bring added value and pertinence to a medical student’s immediate and future career. The process explicitly isolates the need for a student to express and exercise medical reasoning in an organized manner that is not algorithmic or the single-best answer. That is, to provide a reasoning argument for the tests suggested, thus simulating the decision-making processes required by both a referring physician and a radiologist. This interplay is required to provide individualized and effective image-based care, which can be lost in more traditional image interpretation-only-based experiences. Additionally, the strong correlation between understanding how to interpret imaging reports and confidence in discussing future healthcare decisions with patients is a hugely important contribution to all physicians’ education since it is central to their role as referring physicians. Prior to the implementation of the Mystery Case module, radiology instruction at the University of Chicago addressed skills beyond image interpretation such as thoughtful exam ordering and resource allocation. However, it was weighted more towards image interpretation and it wasn’t linked to cases or “real-life” examples where these issues were played out directly in a mock patient care situation.

The experience also emphasizes the importance and value when referring colleagues provide accurate patient history since provided patient history has been shown to influence radiologist imaging interpretation as well as influence what serial imaging is requested for many common disease work-ups [10-13]. Although the most appropriate study for a given clinical question can be found utilizing the ACR Appropriateness Criteria [9], other factors may influence the test ordered, such as the length of time of the imaging study, patient capacity, preparation required prior to imaging, and patient allergies among many others. While knowledge of radiation risks may not alter ordering practices, with CT, for example, it empowers referring physicians to take a role in the discussion and education of what constitutes an appropriate study and how to best inform patients of the benefits and risks of imaging studies [16-17].

Similarly, exam protocols have been developed to achieve optimal imaging quality and diagnostic performance, which, when not followed, may result in nondiagnostic or less sensitive exams for a particular pathology. For example, proper patient preparation, such as fasting prior to positron emission tomography/computed tomography (PET/CT), is paramount, which would otherwise impact sensitivity for cancer detection [18-19]. Improved basic global understanding by referring physicians of radiology exams and how they are best-performed results in better communication with patients and, at the very least, the awareness that calling the radiologist prior to many studies before initiating the exam may be in the best interest of patients. Any improvement in this area has been shown to impact patient satisfaction and health outcomes [20-21]. Stronger referring physician skills can potentially improve imaging throughput, quality of studies obtained, patient care, patient satisfaction, and, ultimately, our relationship as partners in the medical care team.

Limitations

There was no control group for this curriculum. Although responses were overall positive, the Mystery Cases represented a required graded assignment for all medical students. Since this program is in its infancy, it is difficult to measure the long-term effect of this curriculum and whether improvements persist. Understandably, a more robust means of measuring the effectiveness of this module is needed and an Objective Structured Clinical Examination (OSCE), such as those performed during traditional core student clinical clerkships, should be considered. While image ordering and interpreting results for patients are often part of the current set of OSCEs, adding this aspect of evaluation may provide a lens by which to evaluate this curriculum in a more clinical setting. However, the initial results are promising.

## Conclusions

Implementation of this radiology educational module may, in some way, help bridge the gap between the physicians who order radiologic studies and the radiologists who interpret them. Medical students provide an obvious opportunity to exert greater global adoption of these skills, as these students will be responsible for the bulk of future referrals.
